# Multi-acid synergistic fermentation enhances the quality of bran feed

**DOI:** 10.3389/fmicb.2025.1646911

**Published:** 2025-09-02

**Authors:** Wanshu Pan, Binbin Li, Kah Hui Chong, Song Wang, Ling You, Xin Wang, Mahmud Ab Rashid Nor-Khaizura, Nor Afizah Mustapha, Nazamid Saari, Wan Zunairah Wan Ibadullah

**Affiliations:** ^1^Department of Food Science, Faculty of Food Science and Technology, Universiti Putra Malaysia, Serdang, Malaysia; ^2^Faculty of Agriculture, Forestry and Food Engineering, Yibin University, Yibin, China; ^3^Solid-State Fermentation Resource Utilization Key Laboratory of Sichuan Province, Yibin University, Yibin, China; ^4^College of Food Science, Sichuan Agricultural University, Ya’an, China

**Keywords:** Nongxiangxing Baijiu, acid-producing microbiota, multi-acid synergistic fermentation, fermented bran, feed

## Abstract

**Introduction:**

The production of Nongxiangxing Baijiu (Chinese liquor) involves a complex interplay of microbial community metabolism and multi-microbial co-fermentation. The Nongxiangxing Baijiu pit mud is rich in anaerobic acid-producing microorganisms, and this study was designed to investigate the impact of multi-acid synergistic fermentation on feed quality.

**Methods:**

Three Nongxiangxing Baijiu pit muds were subjected to selective serial passage (SSP) three times with four different media (GM, LM, GY, and LY). All samples fermented in GM exhibiting more microbial growth and higher total titratable acidity. Microbial composition analysis of these samples revealed the presence of three acid-producing microbiota (GMAS2, GMBS3, and GMCS3) which were then selected for bran fermentation with three times of SSP.

**Results:**

The bran fermented with acid-producing microbiota was rich in *Pediococcus* and *Lactobacillus* and exhibited increased total titratable acidity and organic acid levels. Electronic nose and organic acid composition analysis revealed that GMAS2S3 (bran fermented with GMAS2 that underwent three times of SSP) had more pronounced flavor characteristics and a higher abundance of acids. Proximate and amino acid analyses confirmed that GMAS2S3 had a higher protein content (22.8%) than the conventional feed (22.8% vs. 16–18%) with abundant amino acids (229.41 mg/g). Palatability evaluation analysis revealed that GMAS2S3-supplemented groups initially showed significantly lower feed intake than the basal diet group, but exceeded basal diet intake during the later adaptation phase.

**Discussion:**

In conclusion, multi-acid synergistic fermentation using anaerobic acid-producing microbiota from baijiu pit mud enhanced bran feed nutritional quality and organic acid content, while maintaining palatability, paving a way for a cost-effective alternative animal feed.

## Graphical abstract

**Figure:**
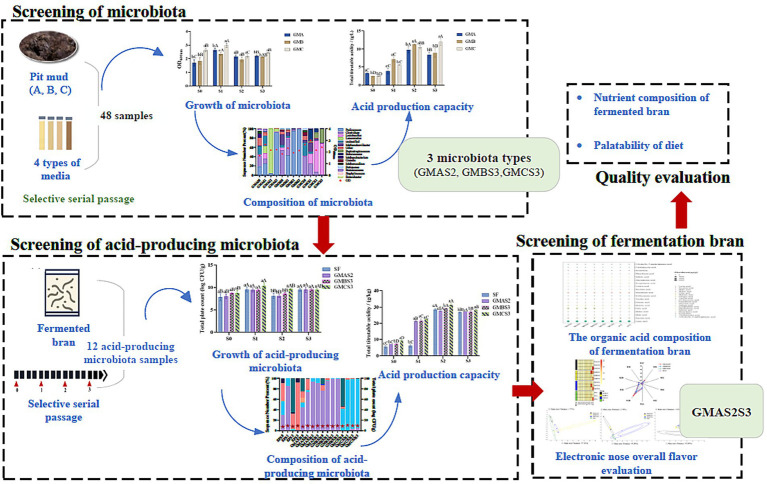
Overview of the experimental design and major findings.

## 1 Introduction

Animal feed is crucial for animal-derived food production and serves as the foundation for the development of the breeding industry (Tolosa et al., 2021). However, with the continued rise in global feed prices, fermentation has been widely used to improve the nutritional value, absorption efficiency, as well as the digestibility of feed (Katu et al., 2025), enhancing nutrient utilization in animals and thus reducing the financial pressure (Dawood and Koshio, 2020). Fermented feed reduces antinutritional factors in feed and improves gut health, immune function, and overall animal growth performance (Sugiharto and Ranjitkar, 2019; Predescu et al., 2024).

In addition to nutritional components such as carbohydrates, proteins, and lipids, the acids in fermented feed also play important roles in animal growth and metabolism. They promote digestion and absorption, enhance nutrient utilization to improve weight gain and feed conversion rates, and ultimately, meat quality (Giorgino et al., 2023; Liu et al., 2023; Wang et al., 2023). Ng and Koh (2017) reported that the acidic compounds in the feed also inhibit the growth of harmful microorganisms, thereby improving the animals’ intestinal health. Furthermore, increasing the acidity in feed is an effective approach to replace antibiotics (Okoye et al., 2023).

The primary origin of acids in animal feed includes: (1) microbial fermentation (Chen et al., 2023; Hu et al., 2023); (2) the addition of acidifiers including inorganic acids such as phosphoric acid, hydrochloric acid, and sulfuric acid, as well as organic acids like formic acid, acetic acid, citric acid, and malic acid (Polycarpo et al., 2017); and (3) naturally occurring acidic substances such as distillers grains, whey, and fruit pomace (Roguski et al., 2023; Song et al., 2023). Fermented feed contains numerous organic acids depending on various factors such as the type and quantity of microorganisms involved, moisture, temperature, and fermentation duration (Cai et al., 2025; Polycarpo et al., 2017). For example, the use of *Bifidobacterium* for corn silage fermentation increases the acetic acid concentration (Huang et al., 2021), whereas *Enterococcus* increases the lactic acid content and decreases acetic acid and butyric acid levels (Marciňáková et al., 2008). Customized fermentation by adding selected microbes affects the composition of organic acids and improves the fermentation quality (Wang et al., 2016; Soundharrajan et al., 2017). Moreover, mixed-culture fermentation (Lu et al., 2020) provides numerous enzymes, potentially enhancing the yield and composition of organic acids (Xu et al., 2011). Therefore, selecting appropriate microbes is crucial for producing a high-quality fermented feed rich in organic acids.

Baijiu is a traditional Chinese distilled liquor, which is typically obtained from grains by solid-state fermentation using Daqu (an essential starter for Chinese baijiu, rich in microbial communities, functional enzyme systems, and flavor precursors), followed by distillation and aging (Ye et al., 2021). Nongxiangxing Baijiu production involves a complex interplay of microbial community metabolism and multi-microbial co-fermentation (Wang S. et al., 2024; Wang Y. et al., 2024; Zhang et al., 2024). The pit mud used in the Baijiu brewing process is a key source of microorganisms, primarily genera such as *Lactobacillus*, *Pediococcus*, *Clostridium*, *Acetobacter*, *Sedimentibacter*, and *Methanobacterium* (Ren et al., 2024), facilitating the production of a variety of short-chain fatty acids (such as acetic acid, propionic acid, lactic acid, butyric acid, and valeric acid), alcohols, aldehydes, esters, and other substances (Zhang Y. et al., 2021). However, these microbes can produce butyric acid, an organic acid that can negatively affect the feed palatability (Galfi and Bokori, 1990), so selecting the appropriate mix of microbes is essential for improving feed quality.

To address the growing need for sustainable and economical feed alternatives in livestock production, the current study was designed to investigate the microbial communities in Baijiu pit mud to establish an effective multi-acid synergistic fermentation system for optimizing organic acids in animal feed. A novel alternative feed was formulated by manipulating microbial community composition to produce beneficial organic acids during fermentation, transforming low-value wheat bran into a high-quality feed ingredient. This research focuses on duck applications due to their high feed conversion efficiency. Although this research primarily targets poultry applications, the fermentation approach has potential applications across various livestock species, such as swine and aquatic animals. The quality of fermented bran feed was comprehensively evaluated to verify its palatability and nutritional level, ensuring a safer and more sustainable feed alternative that promotes animal health while reducing the feeding costs. Future studies will examine meat quality effects and applications in swine and ruminants.

## 2 Materials and methods

### 2.1 Materials

Four different cultural media were used as follows: (1) glucose-based medium (GM) containing 20 g/L of glucose, 2.0 g/L of (NH₄)₂SO₄, 0.5 g/L K₂HPO₄, 10 g/L of peptone, 10 g/L of yeast extract powder, 0.1 g/L of MgSO₄·7H₂O, 0.015 g/L of FeSO₄·7H₂O, 0.01 g/L of CaCl₂, 0.01 g/L of MnSO_4_·H_2_O, 0.002 g/L of COCl₂, and 0.002 g/L of ZnSO_4_, pH 7.0; (2) lactic acid medium (LM) containing 10 g/L of lactic acid, 5 g/L of CH₃COONa, and all constituents as in GM, but without glucose, pH 7.0; (3) glucose + yellow water medium (GY) containing 1 g/L of yellow water and all constituents as in GM, pH 7.0; (4) lactic acid + yellow water medium (LY) containing 1 g/L of yellow water and all constituents as in LM, pH 7.0.

### 2.2 Microbial culture

**Figure 1:**
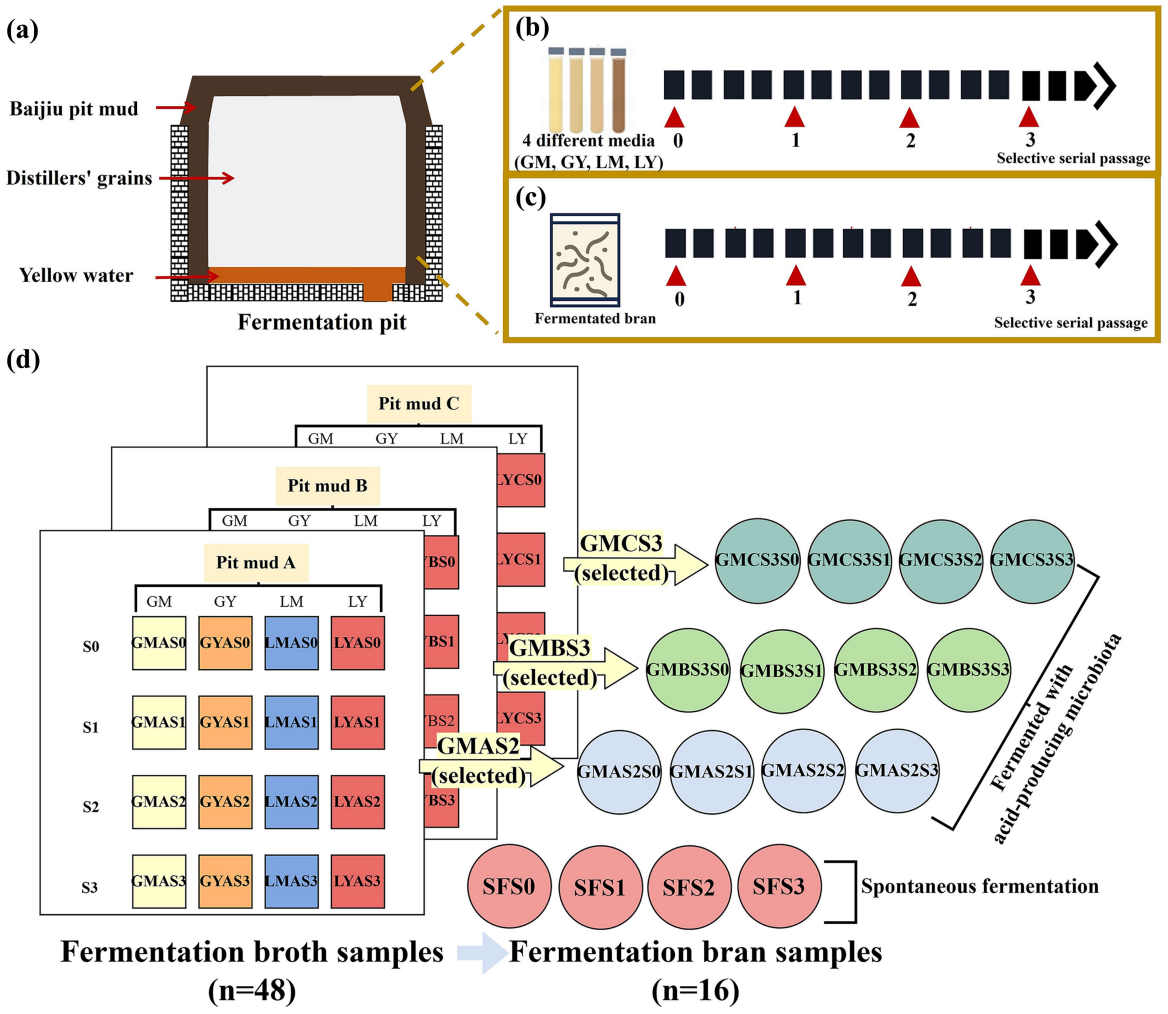
Overview of sample collection, microbial domestication, and samples: **(a)** diagram of the Nongxiangxing Baijiu fermentation pit; **(b)** fermentation broth (GM, GY, LM, and LY) preparation by pit mud microbiota with selective serial passage (SSP) (LM: Lactic acid medium; LY: Lactic acid + yellow water medium; GM: Glucose-based medium; GY: Glucose + yellow water medium); **(c)** bran fermentation by acid-producing microbiota with SSP; **(d)** summary of the fermentation broth samples and fermentation bran samples.

Baijiu pit mud samples (100 g, labeled as A, B, and C, respectively) and yellow water, a nutrient-rich byproduct of the Baijiu fermentation process (Guo et al., 2024), were collected from Sichuan Yibin Gaozhou Liquor Co., Ltd. (Figure 1a). The basal diets consisted of commercially available standard feed purchased from Meishan Shuxia Feed Co., Ltd. (Meishan, China).

Fermentation broth samples (*n* = 48) were obtained through selective serial passage (SSP) of pit muds (A, B, and C) in the different culture media (GM, GY, LM, and LY) (Figure 1b), with acid-producing microbes used to ferment bran samples (*n* = 16) via three SSPs (Figures 1c,d).

Bajiu pit mud samples (30 g) were thoroughly mixed with 600 mL of culture media (referred to as S0, the sample before SSP) and anaerobically cultured at 25 °C for 48 h (referred to as S1, the first cycle of SSP). Fermentation was conducted in a cylindrical glass fermentation vessel (volume 660 mL; 30 cm height × 5.5 cm diameter; manufactured by Kunming Shanglin Plastic Packaging Co., Ltd., Kunming, China) The enriched cultures underwent two subsequent cycles of SSP, with the addition of 30 mL of culture medium in each cycle, designated as microbiota S2 and S3, respectively (Figure 1b). The microbial growth of fermentation broth from each SSP was quantified by measuring the OD_600_ using a UV–Vis spectrophotometer (UV-1500PC, Shanghai Macylab Instrument Co., LTD, Shanghai, China). The total titratable acidity (TTA) was quantified according to GB 12456–2021 (the State Standard of the People’s Republic of China) and determined by titration with 0.1 M NaOH and expressed as lactic acid equivalent. GMAS2 (GMA means the culture of Baijiu pit mud A are from glucose medium, S2 is after two times of selective serial passage.), GMBS3, and GMCS3 were selected for subsequent bran feed fermentation due to the higher TTA and desired bacterial composition.

The impact of three optimal acid-producing microbiotas on bran feed fermentation was evaluated by fermentation with SSP with spontaneous fermentation acting as the control group. Briefly, 324 g of bran was thoroughly mixed with 126 g of purified water and 50 mL of selected fermentation broth before the addition of 1% (w/w) sucrose and 0.5% (w/w) urea (S0). This formulation was based on pilot experiments and the selected fermentation broth was replaced with purified water in the control group. The mixtures were incubated at 25 °C for 72 h using a fermentation bag for the first cycle of SSP (S1), then two subsequent cycles of SSP (S2 and S3) were conducted using 5 g of fermented bran from the previous SSP, 135 g of purified water, 360 g of fresh bran, 1% (w/w) sucrose, and 0.5% (w/w) of urea anaerobically at 25 °C for 72 h for each cycle (Figure 1c).

The total plate counts (TPC) were determined according to GB/T 13093–2023 (National Standards of the People’s Republic of China for Determination of bacterial count in feeds). Briefly, 100 μL of serially diluted homogeneous samples (25 g of sample homogenized in 225 mL of sterile saline solution, followed by 10-fold serial dilutions up to 10^−6^) was plated on nutrient agar and incubated at 36 °C for 48 h. The TPC was reported as colony-forming units per g (CFU/g). The microbiota for bran feed fermentation with the highest TTA, TPC, and desired microbial composition were selected for subsequent analysis.

### 2.3 Microbial composition analysis

Microbial DNA was extracted from the fermentation broth and fermented bran samples using the cetyltrimethylammonium bromide (CTAB) method as described by Ren et al. (2024). The 16S rRNA genes of distinct regions (16S V3-V4) were amplified using specific primers 341F (5′-CCTAYGGGRBGCASCAG-3′) and 806R (5′-GGACTACNNGGGTATCTAAT-3′). The PCR products were separated by 2% agarose gel electrophoresis and purified using the Universal DNA Kit (TianGen, China). Sequencing libraries were generated using NEB Next^®^ Ultra DNA Library Prep Kit (Illumina, United States) and index codes were added according to the manufacturer’s instructions. Library quality was determined using the Agilent 5400 (Agilent Technologies Co. Ltd., United States). Finally, the library was sequenced using an Illumina NovaSeq platform generating 250 bp paired-end reads which were analyzed using Qiime2docs (Yang et al., 2022). The sequence data were analyzed by Wekemo Bioincloud (https://www.bioincloud.tech/) including linear discriminant analysis (LDA), effect size (LEfSe) and principal coordinates analysis (PCoA).

### 2.4 Composition analysis of fermented bran

#### 2.4.1 Organic acid composition

The fermented bran samples (GMAS2S2: bran fermented with GMAS2 that underwent two times of SSP, GMAS2S3, GMBS3S2, GMBS3S3, GMCS3S2, GMCS3S3, and their corresponding spontaneous fermented bran) with the highest TTA and desired microbial composition were subjected to organic acid composition determination using low molecular weight organic acids (LMWOAs) following the method described by Hua et al. (2022). The samples were mixed with 500 μL of methanol/H_2_O/formic acid (30:70:0.1, v/v/v) with two steel balls, vortexed for 60 s and ground at 55 Hz for 1 min before centrifugation for 10 min (12,000 × g, 4 °C). The supernatant was filtered through a 0.22 μm membrane for LC–MS analysis on an ACQUITY Liquid chromatography (Waters, United States) and an AB5000 mass spectrometer (AB SCIEX, United States).

#### 2.4.2 Flavor composition

An electronic nose was used to detect the flavor compounds in the fermented bran samples according to the method described by Lan et al. (2022) and Wang Y. et al. (2024). Briefly, 2 g of fermented bran was placed in a 20 mL headspace vial and the overall aroma was assessed using the PEN3 electronic nose (Beijing Ying Sheng Heng Tai Technology Co., Beijing, China) with a gas flow rate of 300 mL/min, a test time of 120 s, and a cleaning time of 100 s. The sensor information included W1C (aromatic and benzene components), W5S (nitrogen and oxygen compounds), W3C (aromatic and ammonia compounds), WGS (hydrogen selectivity), W5C (short-chain alkanes and aromatic components), W1S (methyl group), W1W (organic sulfide), W2S (alcohols, aldehydes, and ketones), W2W (aromatic and organic sulfides), and W3S (long-chain alkanes). The data were analyzed using WinMuster Software and Device Documentation Version 1.6.2.

### 2.5 Quality evaluation

The selected fermented bran (GMAS2S3) containing a high content of organic acids and a more favorable microbial community was subjected to quality evaluation.

#### 2.5.1 Proximate analysis

The moisture content was determined using the direct drying method in Chinese National Standard GB/T 6435-2014. Crude protein content was analyzed based on the Kjeldahl nitrogen determination described in GB/T 6432-2018. Moreover, the crude fat content was quantified by the Soxhlet extraction method according to GB/T 6433-2006, while the crude fiber content was determined using the acid-alkaline digestion method in GB/T 6434-2006. The crude ash content was assessed based on the high-temperature burning method according to GB/T 6438-2007. The carbohydrate content was calculated based on the following formula: $\text{Carbohydrate content}=100\%-(\begin{array}{l} \text{moisture}\%+\text{crude}\;\mathrm{fat}\%+\\ \text{crude protein}\%+\text{crude}\;\mathrm{ash}\% \end{array})$.

#### 2.5.2 Amino acid quantification and data analysis

Free amino acids were analyzed by HPLC on a 1290 Infinity LC system (Agilent, California, United States) following the method described by Han et al. (2019). Briefly, 60 mg of GMAS2S3 was thoroughly mixed with 450 μL of cold methanol/ acetonitrile/H_2_O (4:4:1, v/v/v) and 16 isotope-labeled internal standards. The mixture was vortexed for 60 s and sonicated at a low temperature for 1 h. The proteins were precipitated at −20 °C for 2 h, collected by centrifugation for 20 min (14,000 × g, 4 °C) and dried in a vacuum centrifuge before subsequent analysis.

#### 2.5.3 Palatability evaluation

The palatability of the fermented bran was evaluated using the method described by Pujaningsih and Mangisah (2020) using Sichuan Shelducks (*Anas platyrhynchos domestica*). A total of 96 ducks (not sexed), with the age (7-week-old), were reared on a farm at Yibin University, Yibin, China at an ambient temperature of 23 °C ± 3 °C and relative humidity of 50–60%. All animal procedures were approved by the Yibin University Animal Care and Use Committee (Approval No. 20231001001) and conducted following National Standard Guidelines for Laboratory Animal-Guideline for Ethical Review of Animal Welfare according to GB/T 35892-2008. Animals received a commercially available standard feed (Meishan Shuxia Feed Co., Ltd., Meishan, China) formulated primarily with soybean meal, corn, rapeseed meal, and wheat middlings. The diet contained crude protein 12.0–16.0%, crude fiber ≤ 10.0%, crude ash ≤ 20.0%, and moisture ≤ 14.0%. Animals had free access to feed and drinking water. The ducks were divided into four feeding groups: three experimental groups (T1: 20% of GMAS2S3 + 80% of basal diet, T2: 40% of GMAS2S3 + 60% of basal diet, T3: 60% of GMAS2S3 + 40% of basal diet) and a control group (T0: 100% of basal diet). Each group had three replicates, with eight ducks per replicate. The palatability test measured the feed intake during days 1–3 (initial response) and days 4–7 (adaptation phase).

### 2.6 Statistical analysis

The data were analyzed using one-way ANOVA in SPSS 23.0 (SPSS Inc., Chicago, IL, United States) with the *post-hoc* Duncan test. Correlations were determined by the R package and a *p*-value < 0.05 was considered significant.

## 3 Results and discussion

### 3.1 Microbial growth, composition, and acid production in different culture media

**Figure 2:**
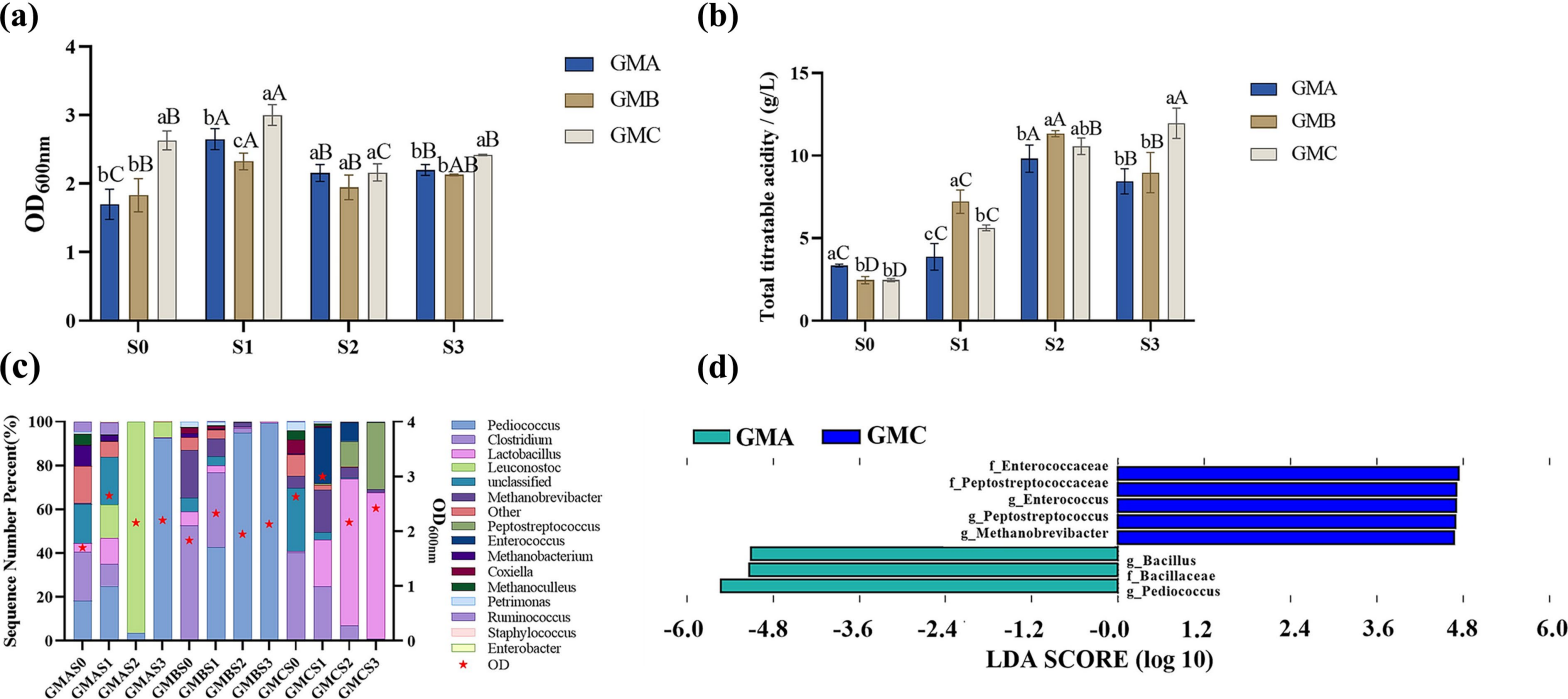
Growth of bacterial communities from Baijiu pit mud samples: A, B, and C cultured on GM medium **(a)**. GM: Glucose-based medium. S0 represents the mixture of the initial Baijiu pit mud and culture media before fermentation. S1 represents the anaerobic fermentation products of S0 at 25 °C for 48 h; S2 and S3 were the selective serial passage (SSP) after the second and third cycles of culture, respectively. Acid production of Baijiu pit mud samples A, B and C in GM during SSP **(b)**. Relative abundance of the bacterial community in the fermentation broth of each pit mud sample **(c)** and the characteristic taxa of GMA and GMC **(d)**. Different letters indicate significant differences between groups (*p* < 0.05): lowercase letters (a–c) denote differences among different groups of samples within the same SSP; uppercase letters (A–D) denote differences among SSP within the same group of samples.

As shown in Figure 2a, the value of OD_600_ of pit mud microbes in GM at each SSP (S1: 2.66 ± 0.34, S2: 2.09 ± 0.12, S3: 2.25 ± 0.15), which has higher S2 and S3 values than GY (S1: 2.81 ± 0.16, S2: 1.74 ± 0.40, S3: 1.64 ± 0.41), LM (S1: 2.90 ± 0.18, S2: 0.35 ± 0.17, S3: 0.05 ± 0.01), and LY (S1: 2.17 ± 0.62, S2: 0.35 ± 0.17, S3: 0.032 ± 0.01) (Supplementary Table S1). This indicates that glucose media was a better culture medium than lactic acid, as glucose is the microbe’s preferred carbon source growth (Kiefer et al., 2002). Furthermore, GM supported greater microbial growth than GY for the three pit mud samples, indicating that yellow water does not effectively promote the growth of pit mud bacteria in selective culture media. This could be due to the presence of soluble starch, organic acids, and more alcohol that disrupt the original carbon-nitrogen balance in selective culture media, causing certain nutrients to be in excess or deficient, thereby inhibiting microbiota growth (Weng et al., 2022).

As shown in Figure 2b, the highest TTA levels were observed in GMAS2 (9.50 ± 0.82 g/L), GMBS2 (11.33 ± 0.19 g/L), and GMCS3 (11.61 ± 0.91 g/L). This elevated TTA with the increased selective serial passages may be due to the microbial growth. A moderate positive correlation was found between microbial biomass (OD value) and TTA (*r* = 0.49) in line with the study of Ke et al. (2022) which reported a correlation coefficient close to 1, suggesting that microbial growth promotes acid production. The lower TTA content in the first cycle might be due to a slower growth rate as the microbiota adapt to the new environment, with the microbes being more efficient at utilizing nutrients in the second and third cycles.

The bacterial community from Baijiu pit muds cultured in GM, with its higher microbial growth and superior acid-producing capabilities, was selected for microbial composition analysis identifying fifteen predominant genera (with relative abundance > 1%) (Figure 2c) including *Pediococcus*, *Clostridium*, *Lactobacillus*, *Leuconostoc*, Unclassified, *Methanobrevibacter*, *Peptostreptococcus*, *Enterococcus*, *Methanobacterium*, *Coxiella*, *Methanoculleus*, *Petrimonas*, *Ruminococcus*, *Staphylococcus*, and *Enterobacter*. LEfSe analysis revealed significant differences in abundance between the pit mud samples (Figure 2d), with the genera *Enterococcus*, *Peptostreptococcus*, and *Methanobrevibacter* being significantly enriched in GMC, while *Bacillus* and *Pediococcus* were significantly enriched in GMA. *Enterococcus*, a common pathogenic microorganism, also serves as a crucial reservoir for antibiotic resistance genes, potentially enabling horizontal gene transfer to other bacteria (Krawczyk et al., 2021). However, SSP in GM led to a significant reduction in the relative abundance of *Enterococcus* across all three pit mud samples, particularly in GMA and GMB, possibly due to the competitive inhibition of dominant microorganisms (*Pediococcus* and *Leuconostoc*). In addition, *Peptostreptococcus* has been linked to pro-inflammatory effects in mice and humans (Chen X. H. et al., 2019; Chen X. W. et al., 2019; Shen et al., 2024) and its relative abundance was reduced in the GMA and GMB pit mud samples after SSP culturing in GM medium. However, due to its high initial abundance in the microbial community of GMC, *Peptostreptococcus* in GMCS3 (30.419%) gradually became the dominant genus during SSP. Furthermore, the abundance of *Staphylococcus* (0.00–0.38%) and *Coxiella* (0.360–6.384%) reduced from S0 to S3 in all samples. This is desirable as *Staphylococcus* and *Coxiella* are detrimental to animal health and production efficiency, and thus negatively impact feed quality (Celina and Cerný, 2022; Ou et al., 2017).

Genera such as *Clostridium* and *Ruminococcus* belonging to the class *Clostridia* (Guo et al., 2020), *Bacteroides* and *Petrimonas* belonging to the class *Bacteroidia* (Ye et al., 2016), and *Methanobrevibacter*, a typical methanogen (Chaudhary et al., 2018), were identified in the pit mud samples. These microbes regulate the ecological balance of pit mud (Hu et al., 2016; Liu et al., 2017). After continuous passage, the different pit mud microbial communities became predominantly acid-producing such as *Pediococcus* in GMA and GMB, and *Lactobacillus* in GMC (Figure 2c).

Although GMBS2 showed superior acid-producing capability compared to GMBS3, it had higher proportions of *Enterococcus* and *Coxiella* known to be detrimental to the host and posing a risk to food safety (Krawczyk et al., 2021). Thus, GMBS3 was selected over GMBS2 for further analysis. Overall, based on the microbial growth, acid-production capability, and microbial community composition results, GMAS2, GMBS3, and GMCS3 were selected for subsequent bran fermentation.

### 3.2 Microbial growth, composition, and acid production capacity of the acid-producing microbes in bran

**Figure 3:**
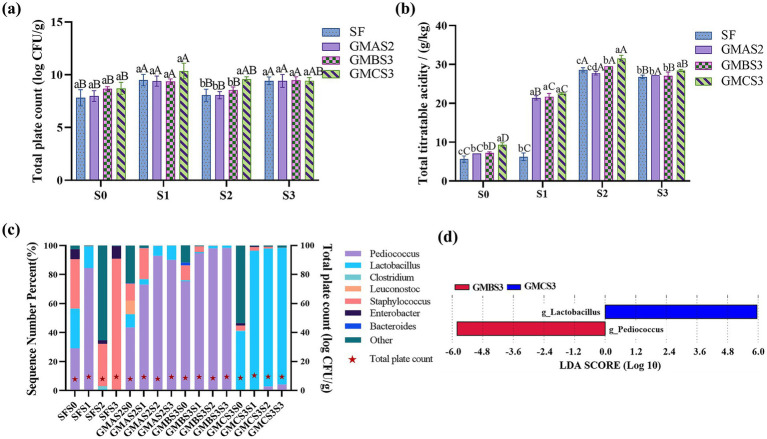
Total plate counts of GMAS2, GMBS3, and GMCS3 **(a)** in fermentation bran during selective serial passage (SSP). S0 is the initial bran sample after inoculation with the acid-producing microbes GMAS2, GMBS3, and GMCS3, respectively. S1, S2, and S3 are the fermentated bran samples after the first, second, and third SSP, respectively. SF is control group of spontaneous fermentation. **(b)** The total titratable acid of the bran samples fermented with GMAS2, GMBS3, and GMCS3 throughout SSP. **(c)** Relative abundance of the acid-producing microbes in the fermentation bran. **(d)** The characteristic taxa of GMBS3 and GMCS3 fermented bran **(d)**. Different letters indicate significant differences between groups (*p* < 0.05): lowercase letters (a–d) denote differences among different groups of samples within the same SSP; uppercase letters (A–D) denote differences among SSP within the same group of samples.

There was no significant difference in TPC among the GMAS2, GMBS3, and GMCS3 (Figure 3a) with the increasing to ideal levels (Yu et al., 2020) after three cycles of SSP (9.42 ± 0.61 log CFU/g, GMBS3S3: 9.47 ± 0.34 log CFU/g, and GMCS3S3: 9.43 ± 0.30 log CFU/g), indicating that the acid-producing microbes are capable of stable growth during bran fermentation.

The acid-producing microbiota fermented bran (APMFB) had a significantly higher TTA (GMAS2S1: 21.40 ± 0.55 g/kg, GMBS3S1: 21.69 ± 0.82 g/kg, and GMCS3S1: 22.50 ± 0.27 g/kg, respectively) compared to spontaneous fermented bran (SFB) (6.24 ± 0.97 g/kg) (Figure 3b), suggesting that the acid-producing microbes adapted more effectively to the bran fermentation environment, exhibiting stable growth and acid production compared to spontaneous fermentation. Acid production increased throughout SSP with a significantly higher TTA in S2 and S3 than in S1.

Seven predominant genera (with relative abundance >1%) were identified in the fermented bran including *Pediococcus*, *Lactobacillus*, *Clostridium*, *Leuconostoc*, *Staphylococcus*, *Enterobacter*, and *Bacteroides* (Figure 3c). The microbial composition of SFB shifted toward an undesirable microbial composition compared to APMFB, as evidenced by the abundance of *Staphylococcus* (90.596%) and *Enterobacter* (8.740%). *Staphylococcus* is a common pathogenic bacterium capable of causing food poisoning when consumed in animal-derived foods (Odetokun et al., 2023). Moreover, *Enterobacter* can adversely affect feed quality by competing to utilize fermentation substrates, increasing protein degradation, and affecting the feed utilization rate (Zhang Q. et al., 2021). Therefore, the presence of these genera in feed fermentation must be controlled to ensure the quality and safety of the final product.

Although the TTA of APMFB and SFB were not significantly different, the former had an increased abundance of acid-producing microbiota, such as *Pediococcus* and *Lactobacillus* (Balakrishnan and Agrawal, 2014). Furthermore, the acid-producing microbial community were stable after multiple passages. Specifically, GMBS3 (GMBS3S1, GMBS3S2, and GMBS3S3) was predominantly enriched with *Pediococcus*, whereas GMCS3 (GMCS3S1, GMCS3S2, and GMCS3S3) was dominated by *Lactobacillus*. This trend was consistent with the microbial composition observed in the corresponding culture media (GMBS3 and GMCS3, respectively). In contrast, GMAS2 fermented bran samples dominated by *Pediococcus* were inconsistent with the fermented broth GMAS2 dominated by *Leuconostoc*. This may be due to *Leuconostoc* not adapting well to the fermented bran environment, and thus was outgrown by *Pediococcus*.

Further LEfSe analysis revealed a significant difference between GMBS3 and GMCS3, as characterized by the dominance of *Pediococcus* and *Lactobacillus* (Figure 3d). *Pediococcus* can produce various organic acids and short-chain fatty acids during milk fermentation (Balakrishnan and Agrawal, 2014). Similarly, *Lactobacillus* can produce organic acids such as lactic acid and acetic acid as well as polypeptide enzymes which enhance the feed quality. These metabolites are environmental modulators that influence the colonization of fermented microbiota and promote the formation of unique microbiota (Ferguson et al., 2010). Thus, optimizing the microbial composition during fermentation to increase the abundance and microbial stability of *Pediococcus* and *Lactobacillus* in fermented bran results in a higher acid content.

### 3.3 Composition of the fermented bran products

#### 3.3.1 Organic acids

**Figure 4:**
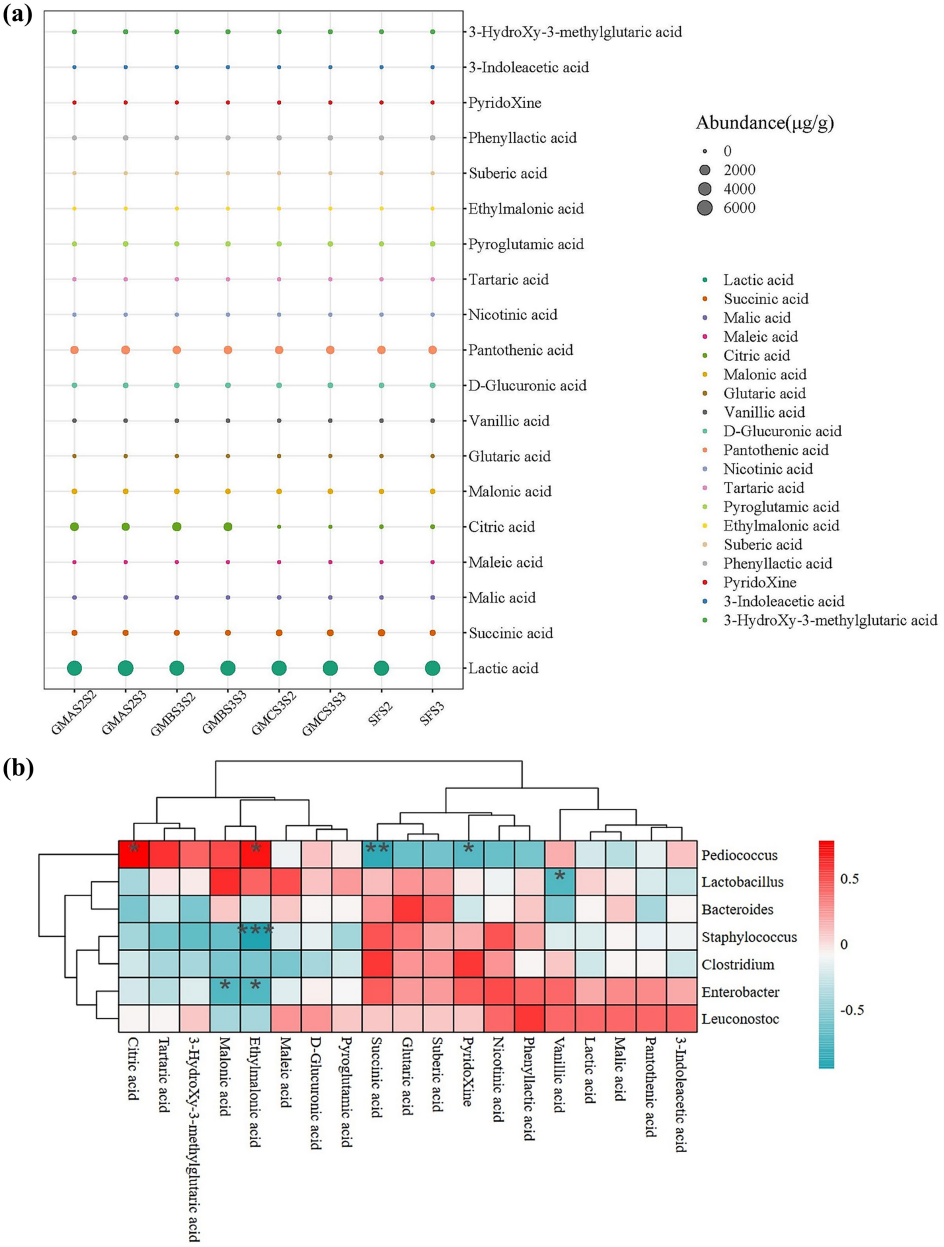
**(a)** Organic acid composition of bran samples fermented with acid-producing microbes at the second (S2) and third (S3) selective serial passage (SSP). **(b)** Correlation between organic acid composition and the microbial composition during bran fermentation. The correlation coefficient (*r*) is displayed in different colors. *p < 0.05, **p < 0.01, ***p < 0.001. SF: spontaneous fermented bran.

Organic acids are often added to animal feed as acidifiers to improve gastrointestinal pH, regulate gut microbial composition, enhance nutrient absorption, and promote individual growth (Tugnoli et al., 2020). APMFB (GMAS2, GMBS3, and GMCS3) at S2 and S3 were selected for organic acid analysis due to the higher TTA and desired microbial composition. Compared to SFB, APMFB contained more lactic acid (5444.59–6244.11 μg/g), citric acid (0–1034.28 μg/g), pantothenic acid (864.99–1053.89 μg/g), pyroglutamic acid (60.45–112.39 μg/g), and phenyl lactic acid (51.52–102.28 μg/g) (Figure 4a) as expected due to the higher TTA. Most organic acids were present at concentrations exceeding 0.1%, a threshold known to influence fermentation (Asriqah et al., 2018; Haq et al., 2017). The citric acid content of bran fermented with GMAS2 (1029.64 ± 173.67 μg/g) and GMBS3 (1124.57 ± 127.68 μg/g) was 47 to 51 times higher than that of the spontaneously fermented group (21.94 ± 9.74 μg/g). Citric acid promotes nutrient absorption and improves poultry carcass quality, and the intestinal microecological environment (Khan et al., 2022). Furthermore, the lactic acid, pantothenic acid, and phenyl lactic acid contents in GMAS2S3 fermented bran were higher than the other samples. In addition, the total organic acid content in APMFB (8050.95 μg/g in GMAS2S2, 8902.82 μg/g in GMAS2S3, 8094.30 μg/g in GMBS3S2, and 8366.91 μg/g in GMBS3S3) was higher than in the SFB (7540.61 μg/g in SFS2 and 7598.11 μg/g in SFS3), indicating that the acid-producing microbiota enhanced the organic acid content of bran feed. GMAS2S3 fermented bran exhibited the highest total organic acid content with a greater acid composition than other groups, indicating its superior feeding potential.

The correlation analysis between organic acid content and the bacterial community revealed that *Pediococcus* was significantly positively correlated with the production of ethylmalonic acid (*r* = 0.714) and citric acid (*r* = 0.786) and negatively correlated with the production of pyridoxine acid (r = −0.714) and succinic acid (*r* = −0.881) (Figure 4b). This is in agreement with the high citric acid production in *Pediococcus*-dominate fermented bran samples (GMBS3S2 and GMBS3S3), as shown in Figures 3c, 4a. In addition, there is a significant negative correlation (*r* = −0.738) between *Lactobacillus* and vanillic acid production, possibly due to the ability of *Lactobacillus* to convert vanillic acid into vanillin (Mostafa and Hashem, 2023), thereby reducing the vanillic acid content in fermented bran feed. Moreover, *Staphylococcus* (*r* = −0.952) and *Enterobacter* (*r* = −0.733) were negatively correlated with ethylmalonic acid production. Furthermore, there was a negative correlation (*r* = −0.733) between *Enterobacter* and malonic acid production which may be due to the antimicrobial effect of malonic acid against harmful microorganisms (Fei et al., 2023).

#### 3.3.2 Flavor evaluation of fermented bran

**Figure 5:**
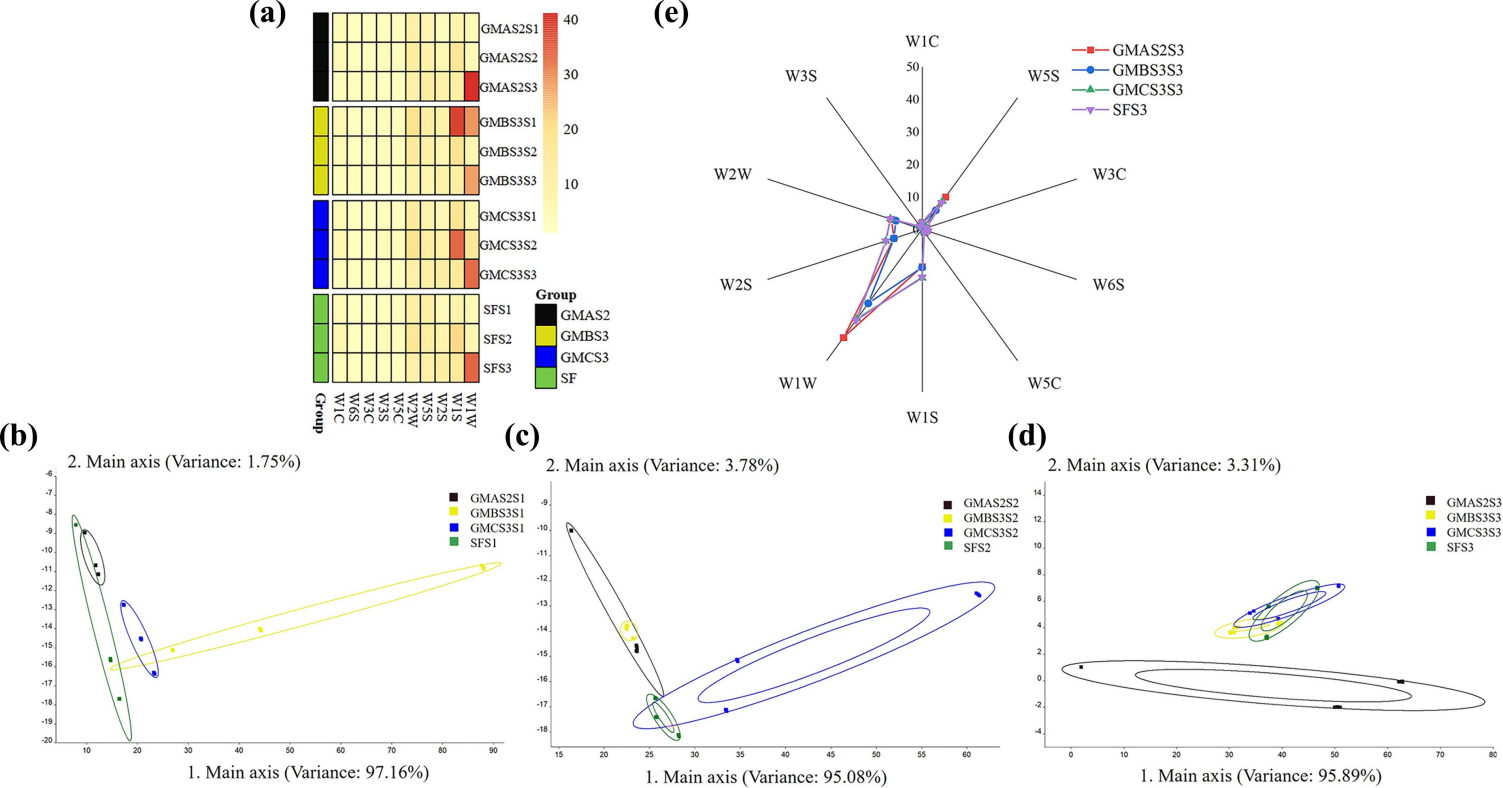
Characterization and analysis of the electronic nose responses: **(a)** sensor response heat map. Principal component analysis (PCA) of the fermented bran samples after the **(b)** first selective serial passage (SSP), **(c)** second SSP, and **(d)** third SSP. SF: spontaneous fermented bran. **(e)** The contour radar chart of the e-nose responses in S3.

The overall aroma of fermented bran was analyzed using an electronic nose (Figure 5), revealing the presence of non-polar organic compounds, aromatic organic compounds, ammonium, short-chain and long-chain alkanes (Kh et al., 2025). In contrast, *Fomitopsis pinicola* inoculation of wheat bran powder was reported to elicit low-level W2W and W2S responses (Tu et al., 2020) suggesting that acid-producing microbiota in this study might synthesize a wider range of substrates. PCA to determine the variations among the samples during the subculturing (Figures 5b–d) revealed that the samples fermented with GMAS2 were distinct from the other samples after three cycles of SSP. Furthermore, the radar plot (Figure 5e) revealed that the bran samples after three cycles of SSP exhibited relatively higher W1S and W1W responses, which correspond to methyl compounds and sulfides, respectively. Notably, GMAS2S3 exhibited higher acid production compared to other samples, possibly associated with its elevated W1W and W5S values. The degradation of proteins and ATP is related to the compounds detected by W1W and W5S, while acids are the byproduct of these processes (Li et al., 2024). The higher response of W1W and W5S in sample GMAS2S3 may have played a key role in driving the increased acid accumulation (Deng et al., 2023).

### 3.4 Quality evaluation of GMAS2S3

#### 3.4.1 Proximate and amino acid composition of GMAS2S3

GMAS2S3 was selected for further nutritional analysis due to its more pronounced flavor characteristics and high acid composition. As shown in Table 1a, GMAS2S3 exhibited a higher protein content (22.8%) and lower fat content (1.8%) than wheat bran (protein: 10.30% and fat: 3.97%) (Lin et al., 2019). The lower fat content in APMFB will not influence the duck’s body weight. This is supported by the fat content of the feed (0.9 to 7.83%) not affecting the body weight and body weight gain of the ducks (Bai et al., 2019). Furthermore, the lower fat content in the feed will prolong its shelf life as it will be less prone to oxidation. In addition, GMAS2S3 had a higher protein content (22.8% vs. 18.50%) compared to that reported by Zhang et al. (2022), possibly due to the enhanced nutrient composition (Márquez-Castillo and Vidal-Quintanar, 2011).

**Table 1:** Nutrient composition of GMAS2S3.

(a) Proximate analysis of fermented feed (*n* = 3, %)					
Moisture	Crude fiber	Crude ash	Crude fat	Crude protein	Carbohydrate
37.2 ± 1.31	30.4 ± 2.39	3.8 ± 1.29	1.8 ± 1.73	22.8 ± 2.14	34.4 ± 2.67

(b) Amino acid content of fermented feed (*n* = 3, mg/g)			
Total essential amino acid = 50.214 mg/g			
Cystine	Isoleucine	Leucine	Lysine
0.003 ± 0.00	1.842 ± 1.17	1.679 ± 0.86	5.214 ± 2.38
Methionine	Phenylalanine	Tyrosine	Valine
1.296 ± 2.04	18.935 ± 0.12	7.401 ± 1.34	13.845 ± 1.71
Total non-essential amino acid = 179.197 mg/g			
Ornithine	Proline	Putrescine	Serine
0.240 ± 0.06	3.156 ± 0.81	0.013 ± 0.01	15.869 ± 2.15
Spermidine	Taurine	Threonine	Tryptophan
0.005 ± 0.00	0.003 ± 0.00	25.313 ± 1.44	0.321 ± 0.07
Alanine	Glycine	Arginine	Asparagine
3.098 ± 1.09	56.607 ± 3.21	0.748 ± 0.13	43.486 ± 0.09
Aminoadipic acid	Choline	Citrulline	Cysteine
0.017 ± 0.01	18.545 ± 0.79	0.021 ± 1.45	0.001 ± 0.00
Glutamate	Glutamine	Histidine	Aspartate
4.507 ± 0.63	0.189 ± 0.10	4.355 ± 1.01	2.697 ± 5.65
Hydroxyproline			
0.004 ± 0.00			

The composition and concentration of amino acids in the feed support the animal’s physiological activities and contribute to the feed quality (Mou et al., 2019). The total amino acid content in GMAS2S3 was 229.41 mg/g, including 50.21 mg/g of essential amino acids and 179.20 mg/g of non-essential amino acids (Table 1b). The free amino acid content in fermented bran was higher than in the fermented bean dregs and soybeans (Heng et al., 2022), suggesting that GMAS2S3 has a good feeding value.

#### 3.4.2 Palatability evaluation

The palatability of the diet containing GMAS2S3 was evaluated based on the daily intake of Sichuan Shelducks (*Anas platyrhunchos domestica*). There was a pronounced decrease in daily intake as the proportion of APMFB in the diet increased (Table 2). During the initial response period (days 1–3), compared to the experimental groups, the control group (T0, 100% basal diet) exhibited the highest daily intake of 202.6 g/head/day, while the T3 group (40% basal diet + 60% GMAS2S3) had the lowest intake of 179.2 g/head/day, with the difference being statistically significant (*p* < 0.05). However, in the adaptation phase (days 4–7), the difference in the feed intake among groups were not statistically significant, and the intake levels in the experimental groups surpassed the control group. Although palatability is not the primary determinant of feed efficacy, maintaining acceptable palatability levels ensures that the feed does not negatively impact intake behavior. In this study, the feed provided enhanced nutrient composition without negatively affecting palatability, which not only supports its practical application but also highlights its potential as a more cost-effective option. While this evaluation is not entirely comprehensive, it provides an preliminary supporting evidence for the practicability of the feed.

**Table 2:** Palatability of diet in ducks containing GMAS2S3.

Treatments (*n* = 5)	Days 1–3 (g/head/day)	Days 4–7 (g/head/day)
T0 (100% basal diet)	202.6 ± 5.03^a^	224.23 ± 6.47^a^
T1 (20% GMAS2S3 + 80% basal diet)	193.4 ± 3.20^b^	229.42 ± 7.33^a^
T2 (40% GMAS2S3 + 60% basal diet)	183.4 ± 2.07^c^	227.53 ± 5.01^a^
T3 (60% GMAS2S3 + 40% basal diet)	179.2 ± 4.02^c^	225.11 ± 5.15^a^

In the same column, means followed by different small letters (a–c) differ significantly (*p* < 0.05).

## 4 Conclusion

Baijiu pit mud was utilized as a source of microbes to enhance the production and composition of acids in fermented bran. The GMAS2S3 bacterial community predominantly comprising *Pediococcus* and *Lactobacillus* after the third passage was selected as the optimal microbe to ferment bran, significantly increasing the total organic acid content with elevated concentrations of lactic acid, pantothenic acid, citric acid, and phenyl lactic acid. Moreover, the GMAS2S3 exhibited abundant nutritional content, with an amino acid profile that meets the physiological requirements of animals, relatively good palatability, and significant feeding value. Future research will focus on assessing the practical value of GMAS2S3 in conjunction with the growth performance of ducks to further explore its potential applications.

## Data Availability

The original contributions presented in the study are publicly available. This data can be found here: NCBI, SAMN50675256-SAMN50675283.
