# Supplementary material for: Multi-acid synergistic fermentation enhances the quality of bran feed
Source: Front Microbiol. 2025 Sep 2;16:1646911. doi: 10.3389/fmicb.2025.1646911 (PMC12436374; doi:10.3389/fmicb.2025.1646911)
Supplement: Supplementary file 1 [file Table_1.docx]

Supplementary Material

# Supplementary Data

Supplementary Table 1. Growth (OD_600nm_) of bacterial communities from Baijiu pit mud samples (A, B, C) cultured on LM (a), LY (b), and GY (c) media.

| 1. **LM medium (n=3, OD_600_)** | | | | |
| --- | --- | --- | --- | --- |
| **Group** | **S0** | **S1** | **S2** | **S3** |
| LMA | 1.57±0.10^bB^ | 2.69±0.26^aA^ | 0.19±0.02^bC^ | 0.05±0.02^aC^ |
| LMB | 2.24±0.35^aB^ | 3.00±0.59^aA^ | 0.34±0.07^bC^ | 0.041±0.01^aC^ |
| LMC | 2.66±0.19^aA^ | 3.000±0.53^aA^ | 0.53±0.13^aB^ | 0.05±0.02^aB^ |
| 1. **LY medium (n=3, OD_600_)** | | | | |
| **Group** | **S0** | **S1** | **S2** | **S3** |
| LYA | 0.95±0.38^cB^ | 2.35±0.37^aA^ | 0.19±0.03^bC^ | 0.02±0.00^aC^ |
| LYB | 1.78±0.30^bA^ | 1.49±0.18^bA^ | 0.32±0.02^bB^ | 0.04±0.01^aB^ |
| LYC | 2.79±0.25^aA^ | 2.69±0.92^aA^ | 0.53±0.09^aB^ | 0.04±0.01^aB^ |
| 1. **GY medium (n=3, OD_600_)** | | | | |
| **Group** | **S0** | **S1** | **S2** | **S3** |
| GYA | 2.19±0.61^aA^ | 2.70±0.69^aA^ | 2.20±0.39^aA^ | 2.10±0.41^aA^ |
| GYB | 2.16±0.25^aA^ | 2.74±0.52^aA^ | 1.44±0.20^bB^ | 1.50±0.27^bB^ |
| GYC | 3.00±0.00^aA^ | 3.00±0.00^aA^ | 1.57±0.17^bB^ | 1.32±0.15^bB^ |

LM: Lactic acid medium; LY: Lactic acid + yellow water medium; GY: Glucose-based medium supplemented with yellow water. S0 represents the mixture of the initial Baijiu pit mud and culture media before fermentation. S1 represents the anaerobic fermentation products of S0 at 25℃ for 48 h; S2 and S3 were the selective serial passage (SSP) after the second and third cycles of culture, respectively. Different letters indicate significant differences (*p*＜0.05): lowercase letters (a-d) for column comparisons, uppercase letters (A-C) for row comparisons.

Supplementary Table 2. Acid production of Baijiu pit mud samples (A, B, and C) in LM (a), LY (b), and GY (c) media

| 1. **LM medium (n=3, g/L)** | | | | |
| --- | --- | --- | --- | --- |
| **Group** | **S0** | **S1** | **S2** | **S3** |
| LMA | 0.50±0.36^bD^ | 1.55±0.91^bB^ | 0.95±1.00^aC^ | 0.32±0.34^aA^ |
| LMB | 2.61±0.33^aA^ | 2.78±0.18^aA^ | 0.20±0.38^bB^ | 0.28±0.53^cB^ |
| LMC | 0.32±0.24^bC^ | 0.15±0.35^cD^ | 0.76±0.38^aA^ | 0.50±0.81^bB^ |
| 1. **LY medium (n=3, g/L)** | | | | |
| **Group** | **S0** | **S1** | **S2** | **S3** |
| LYA | 0.67±0.32^bC^ | 1.55±0.00^bB^ | 1.90±0.33^aA^ | 0.32±0.35^cD^ |
| LYB | 2.96±0.21^aA^ | 3.14±0.47^aA^ | 1.33±0.82^bB^ | 0.67±0.77^aB^ |
| LYC | 0.50±0.34^bB^ | 0.67±0.53^cA^ | 0.15±0.37^cC^ | 0.50±0.81^bB^ |
| 1. **GY medium (n=3, g/L)** | | | | |
| **Group** | **S0** | **S1** | **S2** | **S3** |
| GYA | 3.70±0.12^aB^ | 4.05±0.70^bB^ | 8.88±0.38^aA^ | 8.97±0.18^aA^ |
| GYB | 3.17±0.06^bC^ | 3.70±0.35^bC^ | 9.44±0.38^aA^ | 8.80±0.35^aB^ |
| GYC | 2.46±0.11^cC^ | 7.04±0.77^aAB^ | 7.55±1.13^bA^ | 5.63±0.77^bB^ |

LM: Lactic acid medium; LY: Lactic acid + yellow water medium; GY: Glucose-based medium supplemented with yellow water. S0 represents the mixture of the initial Baijiu pit mud and culture media before fermentation. S1 represents the anaerobic fermentation products of S0 at 25℃ for 48 h; S2 and S3 were the selective serial passage (SSP) after the second and third cycles of culture, respectively. Different letters indicate significant differences (*p*＜0.05): lowercase letters (a-d) for column comparisons, uppercase letters (A-C) for row comparisons.
